# CO_2_ Sensing Characteristics of 2H-MoS_2_-Coated D-Shaped Optical Fiber Sensors

**DOI:** 10.3390/mi17030341

**Published:** 2026-03-11

**Authors:** Han-Mam Kang, Hyung-il Jang, Tae-Jung Ahn, Min-Ki Kwon

**Affiliations:** Department of Photonic Engineering, Chosun University, Gwangju 61452, Republic of Korea; rkdgksaka123@naver.com (H.-M.K.); hyung1_2@naver.com (H.-i.J.)

**Keywords:** MoS_2_ thin films, D-shaped optical fiber sensor, carbon dioxide sensing

## Abstract

In this study, a highly crystalline 2H (hexagonal)-phase MoS_2_ sensing layer with a precisely controlled crystal structure was realized through a combination of DC sputtering and sulfurization annealing processes, and subsequently integrated with a D-shaped optical fiber to develop a highly sensitive carbon dioxide (CO_2_) sensor. Conventionally sputtered MoS_2_ thin films often suffer from the presence of unstable metallic 1T (tetragonal) phases and a high density of sulfur vacancies, which significantly degrade sensor reversibility and long-term stability. Here, high-temperature annealing under a sulfur-rich atmosphere was employed to induce a complete phase transition from the metastable 1T phase to the stable semiconducting 2H phase, while simultaneously healing sulfur vacancies. Enhanced crystallinity was confirmed by Raman spectroscopy. The fabricated sensor exhibited excellent linearity (R^2^ > 0.99) and markedly improved repeatability over a CO_2_ concentration range of 1000–10,000 ppm. This significant performance enhancement is attributed to reversible charge transfer induced by sulfur vacancy passivation, which modulates the complex refractive index of the MoS_2_ layer and optimizes optical interaction with the evanescent field of the D-shaped fiber. The phase engineering and defect-healing strategy presented in this work effectively addresses the drift issues commonly observed in conventional electrical gas sensors and provides a crucial pathway toward the realization of high-performance optical gas sensors.

## 1. Introduction

Global warming and climate change represent some of the most critical environmental challenges facing modern society. As part of global carbon neutrality initiatives, there is an urgent demand for technologies capable of precisely and continuously monitoring atmospheric carbon dioxide (CO_2_) concentrations in real time [1]. Although electronic gas sensors, including semiconductor- and electrochemical-based devices, dominate the current commercial market due to their high sensitivity and fabrication simplicity, they inherently suffer from vulnerability to electromagnetic interference (EMI) and limited long-term stability in harsh chemical environments or hazardous industrial settings with explosion risks [2,3]. In contrast, optical fiber sensors employ light as the signal transmission medium and are therefore completely immune to electromagnetic interference [4]. In addition, they offer unique advantages such as compact size, remote sensing capability over long distances, and intrinsic safety in electrically hazardous environments. Owing to the excellent chemical resistance and thermal stability of silica-based optical fibers, these sensors can maintain high reliability even under corrosive and extreme operating conditions, making them highly attractive as next-generation platforms for environmental monitoring applications [5,6]. To further enhance the sensing performance of optical fiber sensors, extensive research efforts have been devoted to functionalizing the fiber cladding surface with various nanostructured and functional materials [7,8,9]. Among various functional nanomaterials, two-dimensional transition metal dichalcogenides (TMDCs), exemplified by molybdenum disulfide (MoS_2_), have attracted considerable attention owing to their atomically thin structures combined with exceptionally high specific surface areas, which significantly enhance the adsorption probability of gas molecules [9,10,11,12,13]. When a 2D MoS_2_ layer is coated onto the surface of an optical fiber, gas adsorption induces variations in surface charge density through charge transfer interactions. In addition, these changes subsequently modulate the complex refractive index of the MoS_2_ layer, leading to measurable attenuation or phase shifts in the light propagating through the optical fiber. Conventional optical fiber-based gas sensors are often fabricated by chemically etching the cladding to expose the evanescent field and subsequently coating functional nanomaterials onto the etched region. However, such fully etched structures frequently suffer from non-uniform control of the cladding removal depth and mechanical fragility, as the thinned fiber can readily fracture during handling or operation [12]. To overcome these limitations, we employed a D-shaped optical fiber architecture. Unlike approaches that remove the cladding circumferentially, the D-shaped configuration is formed by selectively polishing/etching only one side of the cladding, thereby preserving most of the cylindrical geometry and providing improved mechanical robustness, durability, and etching uniformity [8,14]. As a result, subtle refractive index variations induced by gas molecule adsorption can be sensitively transduced into measurable optical signals, enabling the realization of high-performance gas sensors. However, a critical issue addressed in this study arises from the intrinsic structural defects of MoS_2_ thin films deposited by conventional sputtering processes. Due to the high-energy particle bombardment inherent to sputtering, the resulting films typically exhibit poor crystallinity. Consequently, an undesirable coexistence of the metastable metallic 1T (octahedral) phase and the semiconducting 2H (trigonal prismatic) phase is commonly observed [15,16]. In particular, sulfur vacancies (S-vacancies), which inevitably form during the deposition process, generate a high density of trap sites within the MoS_2_ thin film. These trap states impede charge transport and promote irreversible interactions with gas molecules, thereby severely degrading the recovery characteristics of the sensor [17,18]. A sensor surface containing a high density of the metallic 1T phase and sulfur vacancies may exhibit a rapid initial response upon gas adsorption; however, incomplete desorption of gas molecules during the recovery process leads to a gradual baseline drift. This drift behavior originates from irreversible adsorption and trapped charge states, which prevent full restoration of the sensor to its initial state. Therefore, to realize high-performance CO_2_ sensors with stable and repeatable responses, it is essential to convert the unstable 1T phase into the thermodynamically stable semiconducting 2H phase and to heal sulfur-related defects through sulfur-rich annealing processes [19]. The high crystallinity of the 2H phase enhances charge carrier mobility, thereby improving the signal-to-noise ratio (SNR), while the healed surface enables reversible electron transfer with gas molecules, leading to a marked improvement in sensor reproducibility and long-term stability [20].

In this work, MoS_2_ thin films sputter-deposited on SiO_2_/Si substrates were thermally treated under a sulfur-rich atmosphere to form a highly crystalline 2H-MoS_2_ phase. This process suppresses the metallic 1T phase and compensates sulfur-related defects, thereby optimizing physicochemical interactions with gas molecules. The treated films were then integrated onto the D-shaped optical fiber through a precise transfer process to construct a CO_2_ sensor. Comprehensive structural and chemical characterizations by Raman spectroscopy) were performed before and after phase engineering, together with practical gas sensing evaluations. Ultimately, this study aims to elucidate how sulfur-vacancy suppression promotes reversible adsorption of CO_2_ molecules and how such adsorption dynamics influence the evanescent-field-based optical sensing performance.

## 2. Experimental Methods

### 2.1. Preparation of Sputtered MoS_2_ Film

**Figure 1:**
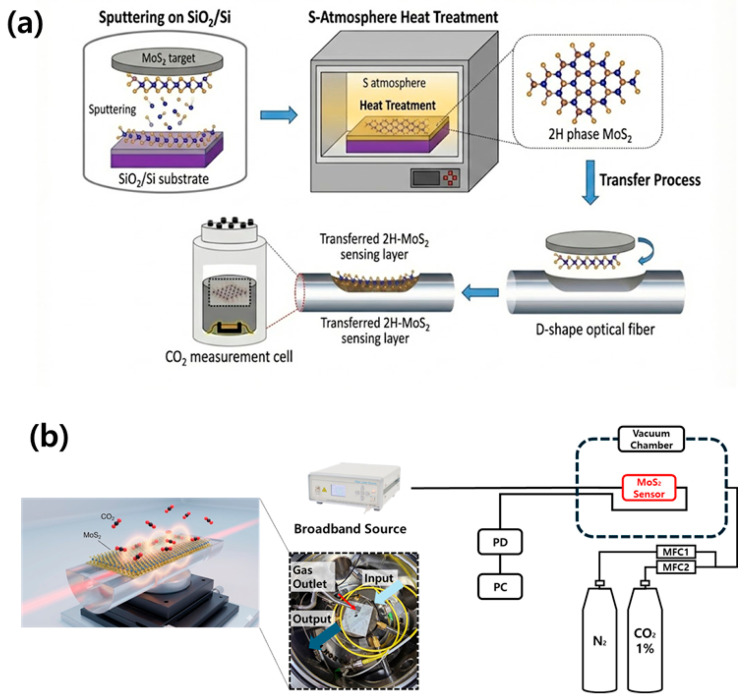
Schematic diagrams illustrating (**a**) the fabrication process of the D-shaped optical fiber sensor and (**b**) the optical measurement setup for CO_2_ sensing. In (**a**), the blue arrows indicate the sequential steps from sputter deposition and sulfur-atmosphere heat treatment to the transfer of the crystalline 2H-MoS_2_ layer onto the D-shaped fiber. In (**b**), the dashed line represents the vacuum chamber environment where gas concentration is regulated by mass flow controllers (MFCs), and the red box highlights the MoS_2_-integrated sensor head within the measurement cell.

The MoS_2_ thin films used as the sensing layer were deposited on SiO_2_/Si substrates by direct current (DC) sputtering. Prior to deposition, a high vacuum below 5 × 10^−5^ Torr was established to remove residual gases inside the chamber. Pre-sputtering was then performed to eliminate surface contaminants from the MoS_2_ target. During the deposition process, a sputtering power of 120 W was applied, and high-purity argon gas (Ar, 50 sccm) was introduced to maintain a working pressure of 5 × 10^−3^ Torr. The substrate temperature was varied from 100 to 400 °C to promote crystal nucleation, and deposition was carried out for 5 min to form the initial MoS_2_ thin films. To suppress the metastable 1T phase and compensate sulfur vacancies inherently generated during sputtering, a N_2_ and sulfur annealing (S-annealing) process was subsequently conducted. The as-deposited samples were transferred to a reaction chamber containing solid sulfur powder and thermally treated at high temperature under a sulfur-rich atmosphere, inducing a phase transition from the 1T structure to the stable 2H phase while simultaneously healing lattice defects. For comparison, reference samples annealed only under a nitrogen (N_2_) atmosphere (700 °C, 1 min) were prepared to evaluate the effect of sulfurization on crystallinity enhancement as shown in Figure 1a. To further stabilize the crystal structure and compensate for sulfur vacancies, the as-deposited MoS_2_ films were subjected to a sulfur-rich annealing process. The sulfurization was performed in a horizontal tube furnace at a peak temperature of 700 °C for a duration of 5 min. High-purity argon (Ar) gas was introduced at a constant flow rate of 100 sccm to facilitate sulfur vapor transport from the upstream zone, where 0.6 g of solid sulfur powder was placed. The working pressure inside the chamber was maintained at 100 Torr throughout the annealing duration. These optimized conditions ensured sufficient sulfur vapor pressure for effective stoichiometric recovery and phase transition.

### 2.2. D-Shaped Optical Fiber Sensor and Electronic Sensors Fabrication

**Figure 2:**
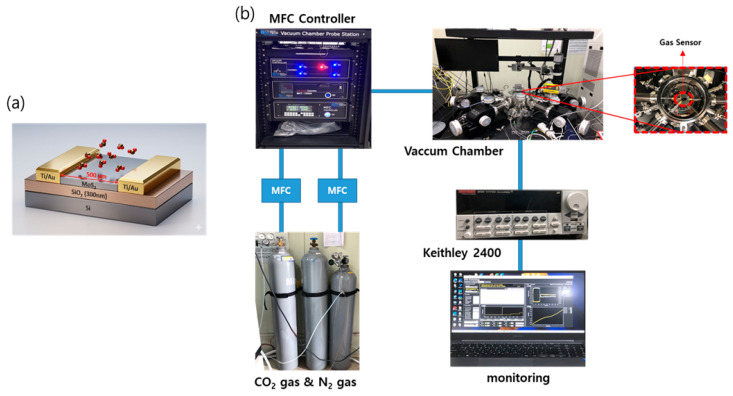
Schematic diagrams illustrating (**a**) the device structure of the resistive-type electronic MoS_2_ sensor and (**b**) the electrical measurement setup for CO_2_ sensing. In (**b**), the blue solid lines represent the gas delivery and electrical signal paths, while the red dashed box and arrows highlight the location of the gas sensor within the vacuum chamber. The Keithley 2400 source meter is connected to the sensor for real-time monitoring of current variations during the measurement for CO_2_ response.

To maximize the exposure of the evanescent field, a D-shaped structure was fabricated by mechanically polishing one side of a single-mode optical fiber (SMF). Partial removal of the cladding layer created a flat polished surface in close proximity to the fiber core, thereby defining an effective optical interaction region for sensing. To integrate the optimized 2H-MoS_2_ thin film onto the D-shaped optical fiber, a poly(methyl methacrylate) (PMMA)-assisted transfer process was employed. PMMA was first spin-coated onto the MoS_2_ thin film, after which the underlying SiO_2_ layer was selectively etched to release the MoS_2_/PMMA composite film from the substrate. Following a cleaning process, the detached film was precisely aligned and laminated onto the polished surface of the D-shaped optical fiber. Subsequently, a low-temperature thermal treatment was applied to enhance interfacial adhesion, and the PMMA supporting layer was removed using acetone, resulting in the completion of the final sensor head. To investigate the CO_2_ sensing behavior of the MoS_2_ thin film, a resistive-type electronic sensor was fabricated, as schematically illustrated in Figure 2. MoS_2_ thin films were first deposited on SiO_2_/Si substrates by DC sputtering. Subsequently, Ti/Au (5/5 nm) electrodes were patterned on the MoS_2_ surface using a shadow mask to define the chemiresistive sensing channel.

### 2.3. Gas Sensing System

As shown in Figure 1b and Figure 2, to evaluate the CO_2_ sensing performance of the fabricated optical fiber sensor, a vacuum chamber-based gas sensing system (M5VC, MSTech, Hwaseong, Republic of Korea ) was constructed. The CO_2_ sensing evaluations were conducted in a high-precision gas sensing chamber (M5VC, MSTech, Hwaseong, Republic of Korea ) with a total volume of 1.5 m^3^. To ensure uniform gas distribution, a constant flow rate of 500 sccm was maintained throughout the measurement cycles. A broadband amplified spontaneous emission (ASE) light source operating in the C-band (ASE-CL-50-B, CiviLaser, Shenzhen, China) was employed as the optical excitation source. The optical output transmitted through the sensor was continuously monitored using a photodetector (S154C, Thorlabs, Newton, NJ, USA) connected to an optical power meter (PM100D, Thorlabs, Newton, NJ, USA), enabling real-time signal acquisition. For an objective performance comparison, a commercial non-dispersive infrared (NDIR) CO_2_ sensor (Cubic CM1107BN, Cubic Sensor and Instrument Co., Ltd., Wuhan, China) and an electrochemical CO_2_ sensor were simultaneously placed inside the same chamber and measured under identical conditions. The CO_2_ concentration was precisely controlled from 1000 to 10,000 ppm in increments of 1000 ppm by using nitrogen (N_2_) as a dilution gas. All measurements were conducted at room temperature (RT). The optical signal attenuation of the fiber sensor and the corresponding voltage responses of the electronic sensors were quantitatively analyzed and directly compared as a function of CO_2_ concentration. The current response of the electronic sensor was analyzed before and after exposure to CO_2_ at a concentration of 40,000 ppm. Electrical measurements were performed using a Parameter Analyzer (Keithley 4200-SCS, Keithley Instruments, Cleveland, OH, USA), enabling precise monitoring of current variations induced by gas adsorption.

## 3. Results and Discussion

**Figure 3:**
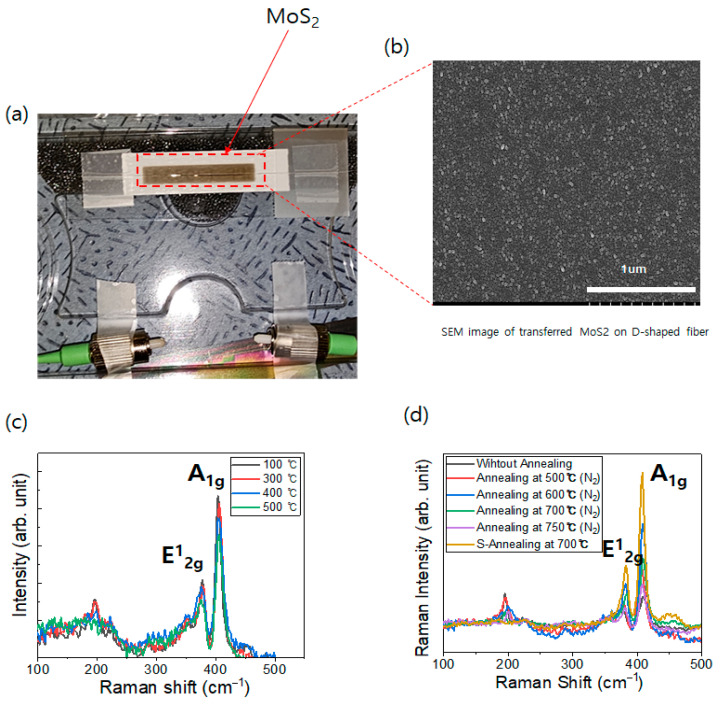
(**a**) MoS_2_-coated D-shaped optical fiber; (**b**) SEM image of the sputter-deposited MoS_2_ thin film; (**c**) Raman spectra of MoS_2_ thin films deposited at different sputtering temperatures; and (**d**) Raman spectra of MoS_2_ thin films before and after annealing.

Figure 3 shows the MoS_2-_coated D-shaped optical fiber sensor. The surface morphology and deposition uniformity of the sputter-deposited MoS_2_ sensing layer were examined using high-resolution scanning electron microscopy (SEM). Unlike solution-based processes or mechanical exfoliation methods, the DC sputtering technique employed in this study produced a highly uniform and continuous film on both SiO_2_/Si substrates and the polished surface of the D-shaped optical fiber as shown in Figure 3b. Such a continuous film morphology is essential to ensure uniform interaction between the evanescent field of the optical fiber and the sensing layer, thereby contributing critically to signal stability and reproducibility. As clearly observed in Figure 3a,b and Supplementary Figure S1, a large-area, continuous MoS_2_ film was successfully and uniformly transferred onto the region above the fiber core. Raman spectroscopy was performed to investigate the crystal structure of the MoS_2_ thin films deposited at different substrate temperatures. As shown in Figure 3c, characteristic in-plane (E^1^_2_g) and out-of-plane (A_1_g) vibrational modes of MoS_2_ are clearly observed at approximately 380 cm^−1^ and 405 cm^−1^, respectively, indicating successful deposition of MoS_2_ thin films via sputtering. In addition, the peak separation between the E^1^_2_g and A_1_g Raman modes exceeds approximately 30 cm^−1^, indicating that the deposited MoS_2_ film possesses a thickness on the approximately 65 ± 15 nm (Supplementary Figure S2). However, an additional Raman feature is detected near 200 cm^−1^, which is commonly associated with the presence of the metallic 1T phase coexisting with the semiconducting 2H phase [17,21]. Although previous studies have reported that increasing growth temperature can promote partial conversion of the 1T phase into the 2H phase, the sputtered films in this work still exhibit residual 1T-related signals, even at elevated deposition temperatures [18]. This behavior can be attributed to differences in sputtering yields between molybdenum and sulfur atoms from the sputtering target. During sputtering, accelerated argon ions preferentially displace sulfur atoms due to their lower atomic mass, leading to sulfur deficiency and local stoichiometric imbalance within the film. Such non-stoichiometry induces lattice distortion, which manifests as 1T-related features in Raman spectra [22]. The metallic 1T phase and sulfur vacancies are known to hinder reversible adsorption–desorption processes of moisture and reactive gas species, thereby degrading sensor response speed, recovery behavior, and overall sensitivity. To verify this effect, both mixed-phase (1T@2H) and phase-pure 2H MoS_2_ thin films were evaluated in terms of their electrical CO_2_ response and optical fiber sensor performance. To eliminate sulfur vacancies and stabilize the 2H crystal structure, an annealing process was introduced. This process proceeds via two dominant mechanisms. First, thermal energy induces atomic rearrangement, driving a complete phase transition from the metastable 1T structure to the thermodynamically stable 2H phase. Second, sulfur atoms supplied from the sulfur-rich atmosphere effectively compensate sulfur vacancies within the lattice, resulting in defect healing. To investigate the elimination of sulfur vacancies through atomic recrystallization induced by high-temperature annealing, thermal treatments were performed under an N_2_ atmosphere while varying the annealing temperature from 500 to 750 °C. As shown in Figure 3d, the intensities of the A_1_g and E^1^_2_g Raman modes gradually increase with increasing annealing temperature, indicating enhanced atomic rearrangement and recrystallization at elevated temperatures. However, despite the improvement in crystallinity, the Raman peak associated with the metallic 1T phase remains clearly observable even after high-temperature N_2_ annealing. This result suggests that thermal energy alone is insufficient to fully suppress the 1T phase and eliminate sulfur-related defects. Therefore, an additional supply of sulfur is required during the recrystallization process to effectively compensate sulfur vacancies. Based on this observation, subsequent annealing was conducted under a sulfur-rich atmosphere to simultaneously promote phase conversion and defect healing. As shown in Figure 3d, Raman spectra after sulfur annealing exhibit the complete disappearance of the 1T-related peak near 200 cm^−1^, accompanied by significantly sharpened and intensified E^1^_2_g and A_1_g peaks, confirming the formation of a highly crystalline 2H-MoS_2_ thin film. The resulting high-quality 2H-MoS_2_ sensing layer is expected to promote reversible charge transfer interactions with CO_2_ molecules, thereby markedly enhancing sensor responsiveness and stability. X-ray photoelectron spectroscopy (XPS) analysis was performed to verify the reduction of sulfur (S) vacancies and the improvement of MoS_2_ crystallinity (Supplementary Figure S3). As illustrated in the spectra, the characteristic peaks of Mo and S were significantly enhanced after the sulfur-rich annealing process. Furthermore, the stoichiometric S/Mo ratio, calculated from the peak area ratios, increased from 1.62 to 2.00 following the treatment. These results confirm that annealing in a sulfur-rich atmosphere is a highly effective method for reducing sulfur defects and restoring the stoichiometry of the sputtered MoS_2_ films.

**Figure 4:**
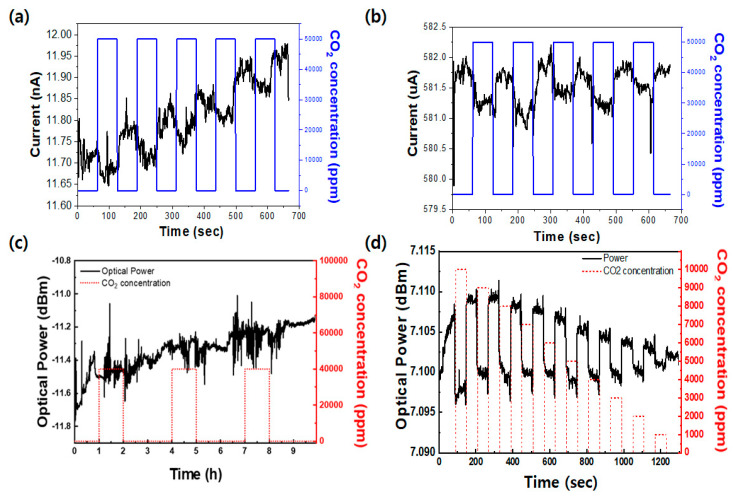
(**a**) Current response of the electronic MoS_2_ sensor before sulfur annealing under CO_2_ exposure (40,000 ppm); (**b**) current response of the electronic MoS_2_ sensor after sulfur annealing under CO_2_ exposure (40,000 ppm); In Figure 4a,b, the black lines represent the measured current, while the blue lines indicate the CO_2_ concentration, (**c**) optical response of the MoS_2-_coated optical fiber sensor before sulfur annealing under CO_2_ exposure (40,000 ppm); and (**d**) optical response of the sulfur-annealed MoS_2_-coated D-shaped optical fiber sensor under various CO_2_ concentrations ranging from 0 to 10,000 ppm.

To investigate the influence of sulfur-annealing-induced crystal structure evolution on CO_2_ sensing behavior, electronic sensors were fabricated using MoS_2_ thin films with mixed 1T@2H and phase-pure 2H crystal structures, and their sensing responses were systematically evaluated. This comparative study was conducted to analyze the intrinsic limitations of electronic sensors and to benchmark their performance against that of optical fiber-based sensors. As shown in Figure 4a,b, the chemiresistor-type electronic sensors exhibit a pronounced dependence of sensing behavior on the structural state of the MoS_2_ thin films. Because reliable sensing responses were difficult to resolve at low CO_2_ concentrations, the sensor performance was initially evaluated at a fixed CO_2_ concentration of 40,000 ppm to clearly assess its intrinsic sensing behavior. In the as-sputtered sensor (1T@2H MoS_2_), although a current response is observed upon CO_2_ exposure, the baseline current fails to recover to its initial level after gas removal, resulting in a severe baseline drift. This behavior is attributed to the high density of sulfur vacancies distributed throughout the film, which act as high-energy trap sites and promote irreversible chemical interactions with CO_2_ molecules. Charges trapped at these defect sites cannot be efficiently released during the desorption process, thereby significantly impairing signal reversibility. For the sulfur-annealed electronic sensor, the conversion of the mixed-phase structure into the semiconducting 2H phase leads to an improvement in the magnitude of the CO_2_ response. However, due to the nanoscale thickness of the conductive channel, substantial electrical noise is introduced by thermal fluctuations on the highly crystalline surface and contact resistance at the electrode–film interfaces [23]. Such electrical noise hinders the precise extraction of small current variations associated with subtle changes in CO_2_ concentration, indicating that the intrinsic advantages of highly crystalline MoS_2_ cannot be fully exploited in electronic sensor configurations. In addition, accurately resolving the sensor response at low CO_2_ concentrations remains challenging due to the limited signal variation. To overcome these limitations, optical fiber-based CO_2_ sensors were fabricated and evaluated, as shown in Figure 4c,d. In the case of the non-annealed MoS_2_ sample, reliable CO_2_ responses were difficult to resolve, which is consistent with the electronic sensor results and can be attributed to suppressed desorption caused by sulfur-vacancy-mediated irreversible adsorption. In contrast, the D-shaped optical fiber sensor incorporating sulfur-annealed MoS_2_ exhibited a remarkable enhancement in sensing performance and excellent operational stability. The highly crystalline 2H-MoS_2_ structure formed through sulfur annealing effectively suppresses sulfur vacancies, which plays a crucial role in ensuring reversible gas adsorption–desorption processes. The defect-minimized 2H-phase surface provides an optimized surface energy landscape that facilitates reversible adsorption and desorption of CO_2_ molecules, thereby maintaining highly consistent and repeatable responses without baseline fluctuation during repeated gas exposure cycles. Notably, because the optical fiber sensor does not rely on direct charge transport measurements but instead exploits light–matter interactions mediated by the evanescent field, it is inherently immune to the electrical noise that critically limits electronic sensors. Stable dielectric property variations associated with the 2H phase are directly converted into optical intensity changes, resulting in a substantially higher signal-to-noise ratio (SNR) and enabling reliable detection of even subtle CO_2_ concentration changes. The observed CO_2_ sensing mechanism can be explained by charge transfer interactions between the MoS_2_ thin film and gas molecules. MoS_2_ exhibits typical n-type semiconductor behavior, while CO_2_ molecules act as electron acceptors. In electronic sensors, adsorption of CO_2_ extracts electrons from the MoS_2_ channel, reducing carrier density and electrical conductivity, which manifests as a decrease in current. In optical fiber sensors, this carrier depletion induces changes in the complex refractive index (n + ik) of the MoS_2_ sensing layer. Variations in carrier concentration affect both the refractive index (*n*) and extinction coefficient (*k*), thereby modulating absorption and scattering of the evanescent field exposed outside the fiber core. Sulfur-annealing-induced phase conversion from the metastable 1T phase to the stable 2H phase, together with effective suppression of sulfur vacancies, significantly enhances the physicochemical stability of MoS_2_ thin films. When integrated with the evanescent field of a D-shaped optical fiber, this strategy overcomes the inherent limitations of electronic sensors and enables the realization of CO_2_ sensors with both high sensitivity and excellent long-term stability. The high-quality 2H-MoS_2_-based D-shaped optical fiber sensor developed in this work demonstrates superior gas responsiveness and recovery characteristics compared with conventional technologies. In contrast, as-sputtered mixed-phase (1T@2H) films inevitably form irreversible bonds with CO_2_ molecules at sulfur-deficient sites due to differences in sputtering yields between molybdenum and sulfur, leading to persistent baseline drift during gas desorption. The optical fiber sensor subjected to sulfur annealing the central strategy of this study clearly demonstrates effective defect healing. Based on the structural stability of the 2H phase, reversible charge transfer interactions with CO_2_ molecules are achieved, resulting in complete baseline recovery without fluctuation even at room temperature.

**Figure 5:**
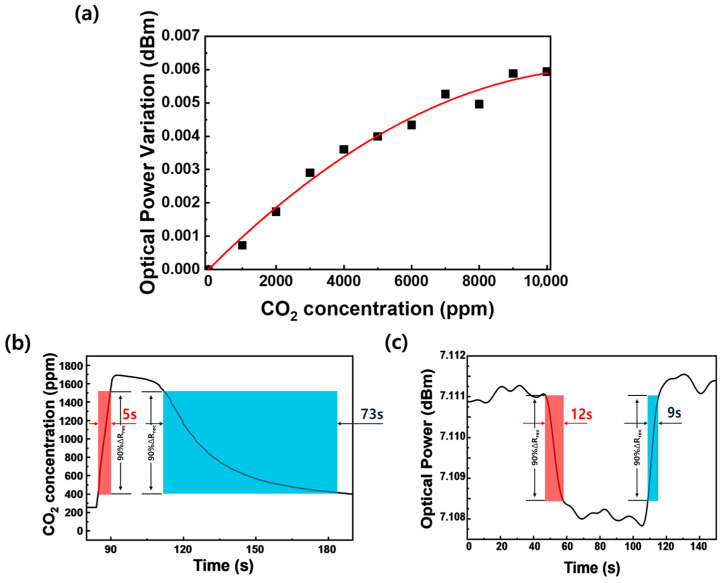
(**a**) Optical output variation of the sulfur-annealed MoS_2_-coated D-shaped optical fiber sensor as a function of CO_2_ concentration. (**b**) Temporal response of a commercial non-dispersive infrared (NDIR) CO_2_ sensor and (**c**) dynamic optical response of the sulfur-annealed MoS_2_-coated D-shaped optical fiber sensor. In (**b**,**c**), the red and blue shaded areas indicate the response time and recovery time, respectively, which are determined by the 90% threshold of the total signal variation.

Figure 5a summarizes the change in optical output as a function of CO_2_ concentration, based on the data obtained from Figure 4d. A monotonic increase in optical signal is observed with increasing CO_2_ concentration, confirming that the proposed sensor responds continuously over a wide concentration range. This concentration-dependent optical modulation indicates that variations in the optical properties of the sensing layer are directly correlated with CO_2_ adsorption, providing a reliable basis for quantitative gas sensing. Figure 5b presents the temporal response of a commercial non-dispersive infrared (NDIR) CO_2_ sensor measured under identical conditions for comparison. Although the NDIR sensor exhibits a detectable response upon CO_2_ exposure, a pronounced delay in both response and recovery is clearly observed. In particular, the recovery process is significantly prolonged, requiring several tens of seconds for the signal to return toward its baseline after CO_2_ removal. This slow recovery behavior originates from the diffusion-limited sensing mechanism of NDIR sensors, in which gas exchange within the optical chamber governs the overall response dynamics. In contrast, the sulfur-annealed MoS_2_-coated D-shaped optical fiber sensor exhibits a comparable response time and a significantly faster recovery behavior compared to conventional NDIR technology as shown in Figure 5c. The optical signal returns to its baseline within only 9 s after CO_2_ removal, demonstrating near-complete and fluctuation-free recovery even under repeated gas exposure cycles. This rapid recovery is a direct consequence of reversible charge transfer interactions between CO_2_ molecules and the highly crystalline 2H-MoS_2_ sensing layer, enabled by effective sulfur-vacancy healing.

The dramatic difference in recovery time highlights a critical advantage of the proposed optical fiber sensor over conventional NDIR technology. While NDIR sensors inherently suffer from slow desorption and gas exchange processes, the evanescent-field-based sensing mechanism allows immediate transduction of adsorption and desorption events into optical signals without reliance on bulk gas diffusion. As a result, the proposed sensor achieves a substantially higher signal-to-noise ratio (SNR) and enables reliable detection of rapid CO_2_ concentration fluctuations, which is particularly advantageous for real-time environmental monitoring and industrial safety applications.

## 4. Conclusions

In this study, a high-performance D-shaped optical fiber CO_2_ sensor was successfully realized through precise control of the crystal structure and defect states of sputter-deposited MoS_2_ thin films. The key achievements of this work can be summarized as follows. First, a sulfur annealing process was introduced to suppress the metastable 1T phase formed during the initial sputtering stage and to induce a complete phase transition into the thermodynamically stable 2H phase. Raman spectroscopy analyses confirmed a substantial enhancement in crystallinity, which provides a fundamental basis for achieving stable and reliable optical signal transduction. Second, defect compensation via sulfur-vacancy healing was demonstrated to be critical for maximizing sensor reversibility. The defect-suppressed 2H-MoS_2_ surface offers an optimal environment for reversible adsorption of CO_2_ molecules acting as electron acceptors, thereby effectively eliminating baseline drift a persistent limitation of conventional electronic gas sensors. Third, the fabricated optical fiber sensor exhibited excellent linearity and reproducibility over a wide CO_2_ concentration range from 1000 to 10,000 ppm. This performance originates from charge transfer induced by gas adsorption, which modulates the complex refractive index of the MoS_2_ sensing layer and precisely controls the attenuation of the evanescent field propagating along the D-shaped optical fiber. In conclusion, the phase engineering and defect-healing strategy presented in this work represents a significant technological advancement toward overcoming the commercialization barriers of two-dimensional-material-based optical sensors. The proposed approach is expected to contribute to the development of highly sensitive and stable environmental gas monitoring systems for a wide range of practical applications.

## Data Availability

The data presented in this study are available on request from the corresponding author.

## Supplementary Materials

The following supporting information can be downloaded at: https://www.mdpi.com/article/10.3390/mi17030341/s1. Figure S1: SEM image of the sputter-deposited MoS_2_ thin film on a D-shaped optical fiber, showing surface morphology and uniformity. Figure S2: (a–d) Cross-sectional SEM images of sputter-deposited MoS_2_ thin films with various deposition times (5, 15, 30, and 60 min) on D-shaped optical fibers, and (e) measured thickness of the sputtered MoS_2_ films as a function of deposition time. Figure S3: XPS spectra of (a) Mo 3d and (b) S 2p orbitals for MoS_2_ thin films with and without sulfur (S) annealing, demonstrating stoichiometric recovery and defect healing.

## Abbreviations

The following abbreviations are used in this manuscript:

MoS_2_	Molybdenum disulfide
2H	Hexagonal
1T	Tetragonal
CO_2_	Carbon dioxide

## References

[1] Jarvis A., Forster P.M. (2024). Estimated human-induced warming from a linear temperature and atmospheric CO_2_ relationship. Nat. Geosci..

[2] Kang H.H.Y., Jung H.T. (2024). Gas Sensors for Climate Change. ACS Sens..

[3] Ughade Y., Mehta S., Patel G., Gowda R., Joshi N., Patel R. (2025). Progress in CO_2_ Gas Sensing Technologies: Insights into Metal Oxide Nanostructures and Resistance-Based Methods. Micromachines.

[4] Butt M.A. (2024). Topic Editorial on Fiber-Optic Sensors. Micromachines.

[5] Khonina S.N., Kazanskiy N.L., Butt M.A. (2023). Optical Fibre-Based Sensors-An Assessment of Current Innovations. Biosensors.

[6] Pendao C., Silva I. (2022). Optical Fiber Sensors and Sensing Networks: Overview of the Main Principles and Applications. Sensors.

[7] Qu W.W., Chen Y.X., Liu S.Q., Luo L. (2025). Advances and Prospects of Nanomaterial Coatings in Optical Fiber Sensors. Coatings.

[8] Del Villar I., Zubiate P., Zamarreño C.R., Arregui F.J., Matias I.R. (2017). Optimization in nanocoated D-shaped optical fiber sensors. Opt. Express.

[9] Zhang H.X., Zhou X., Li X.G., Gong P.Q., Zhang Y.A., Zhao Y. (2023). Recent Advancements of LSPR Fiber-Optic Biosensing: Combination Methods, Structure, and Prospects. Biosensors.

[10] He Y.Q., Xu H.M., Zhang J.D., Zheng D., Zhang G., Fan X.Z., Ou-Yang H., Liu Y.Q., Lv A.C., Zhao J.W. (2025). Molybdenum Disulfide Induced Phase Control Synthesis of Multi-dimensional Co_3_S_4_-MoS_2_ Heteronanostructures via Cation Exchange. Angew. Chem. Int. Ed..

[11] Acosta S., Quintana M. (2024). Chemically Functionalized 2D Transition Metal Dichalcogenides for Sensors. Sensors.

[12] Owji E., Mokhtari H., Ostovari F., Darazereshki B., Shakiba N. (2021). 2D materials coated on etched optical fibers as humidity sensor. Sci. Rep..

[13] Yap S.H.K., Chan K.K., Yeh C.H., Chien Y.H., Yong K.T. (2021). Two-Dimensional MoS_2_ Nanosheet-Functionalized Optical Microfiber for Room-Temperature Volatile Organic Compound Detection. ACS Appl. Nano Mater..

[14] Ying Y., Si G.Y., Luan F.J., Xu K., Qi Y.W., Li H.N. (2017). Recent research progress of optical fiber sensors based on D-shaped structure. Opt. Laser Technol..

[15] Gao B., Du X.Y., Ma Y.M., Li Y.X., Li Y.H., Ding S.J., Song Z.X., Xiao C.H. (2020). 3D flower-like defected MoS_2_ magnetron-sputtered on candle soot for enhanced hydrogen evolution reaction. Appl. Catal. B-Environ..

[16] Kumar R.R., Gupta S., Anbalagan A.K., Khan A., Tai N.H., Lee C.H., Lin H.N. (2025). Time-Dependent Growth of Sputtered MoS_2_ Films on ZnO Nanorods for Enhanced NO_2_ Sensing Performance. Micromachines.

[17] Hong G.S., Kim M.E., Lee J.S., Kim J.Y., Kwon M.K. (2024). Respiration Monitoring Using Humidity Sensor Based on Hydrothermally Synthesized Two-Dimensional MoS_2_. Nanomaterials.

[18] Kim E., Kim J.Y., Kwon M.K. (2023). Synthesis of MoS_2_ Using Chemical Vapor Deposition and Conventional Hydrothermal Methods: Applications to Respiration Sensing. Appl. Sci..

[19] Fu X.Q., Qiao Z.R., Zhou H.Y., Xie D. (2024). Defect Engineering in Transition Metal Dichalcogenide-Based Gas Sensors. Chemosensors.

[20] Parichenko A., Huang S.R., Pang J.B., Ibarlucea B., Cuniberti G. (2023). Recent advances in technologies toward the development of 2D materials-based electronic noses. TrAC-Trend Anal. Chem..

[21] Liu Z.P., Gao Z.C., Liu Y.H., Xia M.S., Wang R.W., Li N. (2017). Heterogeneous Nanostructure Based on 1T-Phase MoS_2_ for Enhanced Electrocatalytic Hydrogen Evolution. ACS Appl. Mater. Inter..

[22] Santoni A., Rondino F., Malerba C., Valentini M., Mittiga A. (2017). Electronic structure of Ar+ ion-sputtered thin-film MoS_2_: A XPS and IPES study. Appl. Surf. Sci..

[23] Fadil D., Sharma J., Rizu M.I., Llobet E. (2024). Direct or Indirect Sonication in Ecofriendly MoS_2_ Dispersion for NO_2_ and NH_3_ Gas-Sensing Applications. ACS Omega.

