# Supplementary material for: CO2 Sensing Characteristics of 2H-MoS2-Coated D-Shaped Optical Fiber Sensors
Source: Micromachines (Basel). 2026 Mar 11;17(3):341. doi: 10.3390/mi17030341 (PMC13028271; doi:10.3390/mi17030341)
Supplement: Supplementary file 1 [file micromachines-17-00341-s001.zip › micromachines-4167499-supplementary.pdf]

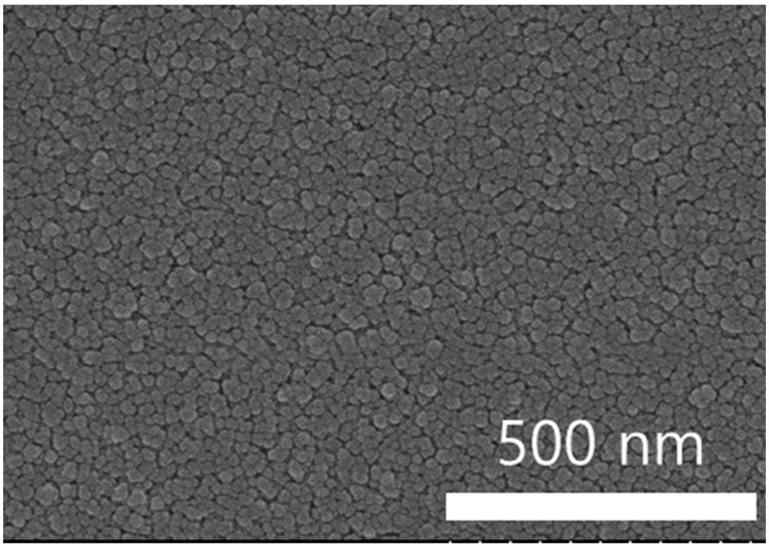

Supplementary figure S1. SEM image of the sputter-deposited MoS_2 thin film on D-shaped optical fiber

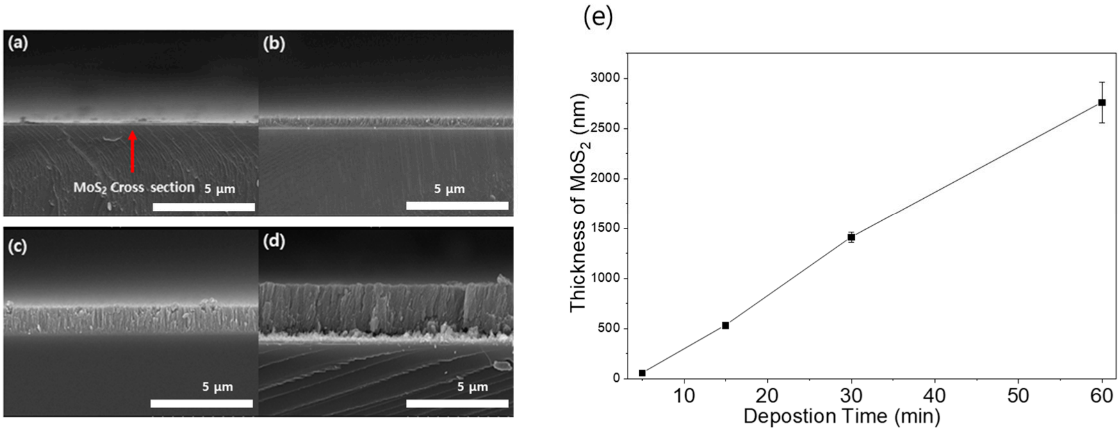

Supplementary figure S2. Cross-sectional SEM image of the sputter-deposited MoS_2 thin film with various depostion time of (a) 5 min, (b) 15 min, (c) 30 min, (d) 60 min on D-shaped optical fiber and Measured thickness of the sputtered MoS_2 films as a function of deposition time

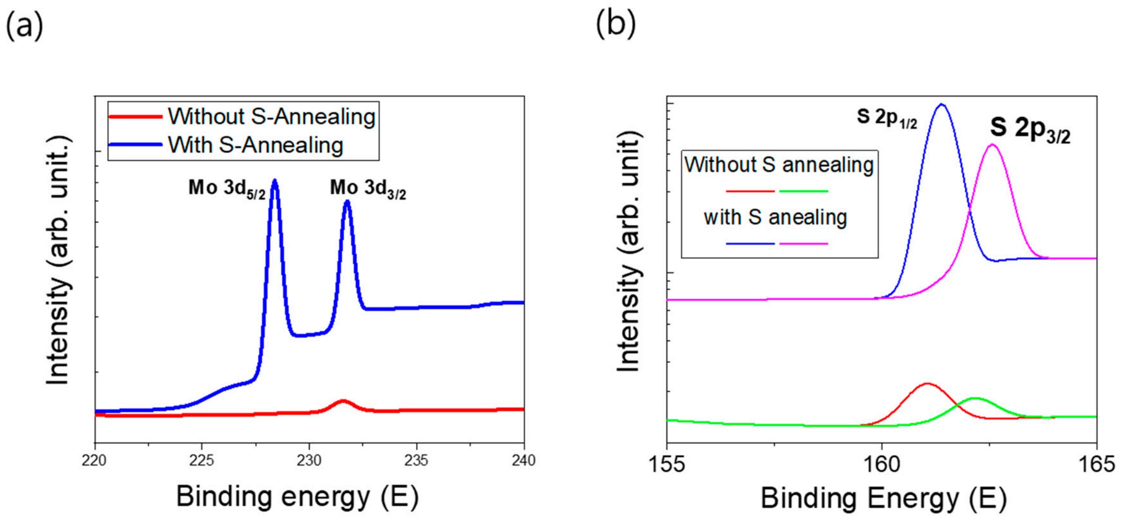

Supplementary figure S3. XPS spectra of (a) Mo and (b) S on MoS2 with and without S annealing.
